# The Impact of the Stringent Response on TRAFAC GTPases and Prokaryotic Ribosome Assembly

**DOI:** 10.3390/cells8111313

**Published:** 2019-10-24

**Authors:** Daniel J. Bennison, Sophie E. Irving, Rebecca M. Corrigan

**Affiliations:** The Florey Institute, Department of Molecular Biology and Biotechnology, University of Sheffield, Sheffield S10 2TN, UK; djbennison1@sheffield.ac.uk (D.J.B.); seirving1@sheffield.ac.uk (S.E.I.)

**Keywords:** ribosome, GTPase, stringent response, ppGpp, assembly cofactors, TRAFAC

## Abstract

Many facets of ribosome biogenesis and function, including ribosomal RNA (rRNA) transcription, 70S assembly and protein translation, are negatively impacted upon induction of a nutrient stress-sensing signalling pathway termed the stringent response. This stress response is mediated by the alarmones guanosine tetra- and penta-phosphate ((p)ppGpp), the accumulation of which leads to a massive cellular response that slows growth and aids survival. The 70S bacterial ribosome is an intricate structure, with assembly both complex and highly modular. Presiding over the assembly process is a group of P-loop GTPases within the TRAFAC (Translation Factor Association) superclass that are crucial for correct positioning of both early and late stage ribosomal proteins (r-proteins) onto the rRNA. Often described as ‘molecular switches’, members of this GTPase superfamily readily bind and hydrolyse GTP to GDP in a cyclic manner that alters the propensity of the GTPase to carry out a function. TRAFAC GTPases are considered to act as checkpoints to ribosome assembly, involved in binding to immature sections in the GTP-bound state, preventing further r-protein association until maturation is complete. Here we review our current understanding of the impact of the stringent response and (p)ppGpp production on ribosome maturation in prokaryotic cells, focusing on the inhibition of (p)ppGpp on GTPase-mediated subunit assembly, but also touching upon the inhibition of rRNA transcription and protein translation.

## 1. Introduction

Widely regarded as one of the most intricate macromolecular assemblies within the cell, the 70S prokaryotic ribosome is essential for the translation of messenger RNA (mRNA) into primary amino acid sequence during protein synthesis. Owing perhaps to its complexity, assembly of the 70S is extraordinarily well controlled in order to maintain high translational accuracy. The 70S ribosome particle is made up of two individual subunits, the small 30S and the large 50S subunits. The *Escherichia coli* 30S consists of 21 ribosomal proteins (r-proteins) along with a single 16S ribosomal RNA (rRNA), while the 50S subunit consists of 34 r-proteins and two rRNAs—the 23S and the 5S [1]. Despite a high level of control over the pathway, 70S assembly is highly modular and, thus, capable of being carried out via a number of parallel pathways with the help of assembly cofactors. These cofactors include RNA/protein-folding chaperones, RNA helicases, RNA/protein modification enzymes and ribosome associated GTPases (RA-GTPases) [2,3].

Within the P-loop NTPase superfamily, GTPases make up a monophyletic superclass that can be further divided into two subclasses that differ in sequence signatures and functional components. The first of these subclasses are the SIMIBI (Signal Recognition Particle, MinD and BioD) GTPases that are involved in protein trafficking, membrane transport and chromosome partitioning [4]. The second subclass are the TRAFAC (Translation Factor Association) GTPases, associated with all aspects of translation, cellular motility, intracellular transport and signal transduction [4]. TRAFAC GTPases are more functionally diverse than the SIMIBI family and many fall under the umbrella of RA-GTPases—a term used to refer to any GTPase involved in ribosome assembly, translation or any other ribosomal process. These GTPases are reported to have an affinity for GTP in the mid-micromolar range [5], although RA-GTPases with much higher affinities for GTP have been identified [6], whereas many Ras-like GTPases have affinities within the low micromolar to picomolar range [5]. This is significant as the physiological concentration of GTP in the bacterial cytoplasm is around 200–500 μM [7], thus enabling RA-GTPases to respond to small fluctuations in the cellular energy state in order to regulate energy-intensive processes such as ribosome assembly and translation.

Many TRAFAC RA-GTPases are important for the correctly timed assembly of both early and late-stage r-proteins onto the mature rRNA [8]. Often considered a checkpoint of accurate ribosome assembly, the purpose of these GTPases is to bind a specific, immature section of the ribosome precursor in the GTP-bound state, hence preventing the association of downstream r-proteins prior to the correct conformation of the RA-GTPase binding site [8]. Maturation of the section in question then stimulates the enzymatic GTPase activity and subsequent dissociation of the RA-GTPase from the ribosome, removing the steric hindrance and allowing further subunit assembly [5].

During conditions of intense conditional stress, the transcriptome and proteome of bacteria are altered to facilitate survival using a signalling pathway termed the stringent response. This pathway was first discovered through investigation of the response of *E. coli* to amino acid starvation. The starved *E. coli* cells produced two nucleotides that appeared as ‘magic spots’ on a chromatograph and were later identified as the nucleotides ppGpp and pppGpp, which function as the effectors of cellular change [9]. Across bacteria, amino acid starvation appears to be a common trigger of the stringent response because amino acids are universally required for growth. However, it is also activated by additional key stressors, such as antibiotics, iron limitation, immature tRNAs and fatty acid limitation [10,11,12,13,14]. There are three groups of proteins responsible for the synthesis and hydrolysis of (p)ppGpp in the cell: small alarmone synthetases (SASs), small alarmone hydrolases (SAHs) and long RelA/SpoT homologues (long-RSHs) [15,16]. These groups are members of the RelA/SpoT homologues (RSH) superfamily, named after the two (p)ppGpp synthesis enzymes found in *E. coli*, RelA and SpoT [15]. ppGpp and pppGpp are synthesised by the transfer of a pyrophosphate group from ATP to the ribose 3′-OH of GDP or GTP, respectively, by nucleophilic substitution [17,18]. Hydrolysis of (p)ppGpp releases the pyrophosphate group and either GDP or GTP.

The stringent response is characterised by an accumulation of up to 2 mM (p)ppGpp, which results in a general reduction in transcription, particularly in genes involved in biosynthesis of macromolecules [19]. Translation is also reduced during the stringent response as a result of an inhibition at multiple points along the protein production route, including a reduction in rRNA transcription, impaired ribosome maturation and an inhibition of translation from mature ribosomes [6,20,21,22]. Ultimately, the impact of the stringent response on ribosome assembly and protein production contributes to slowed growth, which has now been implicated in various bacterial processes such as virulence, biofilm formation and sporulation [23,24]. Here we review the impact of (p)ppGpp on protein production with in-depth coverage of the role of (p)ppGpp in regulating ribosomal biogenesis via its interactions with a subset of RA-GTPases, followed by a brief overview of the inhibition of both the transcription of rRNA genes and the enzymes involved in protein translation by (p)ppGpp.

## 2. A Brief Overview of Ribosome Assembly

The *E. coli* 70S ribosome is a 2.3 MDa complex with a diameter of 210 Å [25]. Up to 70,000 70S ribosomes are estimated to be present within a single *E. coli* cell, constituting between 40%–50% of the cellular dry weight when dividing [26,27]. The ribosome is a ribozyme consisting of roughly 1/3 protein and 2/3 rRNA, which can be further broken down into the 30S small and 50S large subunits. The 30S ribosome is responsible for binding and decoding the mRNA by recognising the complementary base interactions between the mRNA codon and the transfer RNA (tRNA) anticodon [28]. The 50S subunit houses the peptidyltransferase centre (PTC) machinery within domain V of the 23S rRNA and confers amino acid residues onto the novel peptide chain [28]. The PTC is made up of an aminoacyl site (A-site), peptidyltransferase site (P-site) and an exit site (E-site). In addition, the 50S can also prevent premature peptide hydrolysis and the exit tunnel quaternary conformation can aid in the co-translational folding of the nascent chain [29].

The assembly of prokaryotic ribosomes involves the coordinated action of a number of steps, including: rRNA transcription, with subsequent modifications such as pseudouridylation and methylation; r-protein transcription, translation and modification; correct folding and association of both rRNA with r-proteins and the binding and dissociation of biogenesis cofactors. This is thought to proceed via two major strategies: co-transcriptional assembly and rRNA folding-guided assembly [30]. Electron microscopy images revealed the near immediate association of constitutively expressed r-protein complexes to nascent rRNA chains upon transcription by RNA Polymerase (RNAP) [31]. The second strategy suggests that ribosome assembly is limited by RNA folding and that upon correct secondary conformation the relevant r-proteins can associate and stabilise the RNA [32,33]. This folding of rRNA can occur either in a protein-dependent or protein-independent manner [32], mediated by assembly factors which often use GTPase activity as a mechanism of dissociation from the ribosome following correct maturation (reviewed extensively in [5,8,34,35,36,37,38,39]). In agreement with this, the 30S subunit is thought to assemble from the 5′–3′ of the 16S rRNA, in line with the orientation of transcription and subsequent folding [40].

**Figure 1:**
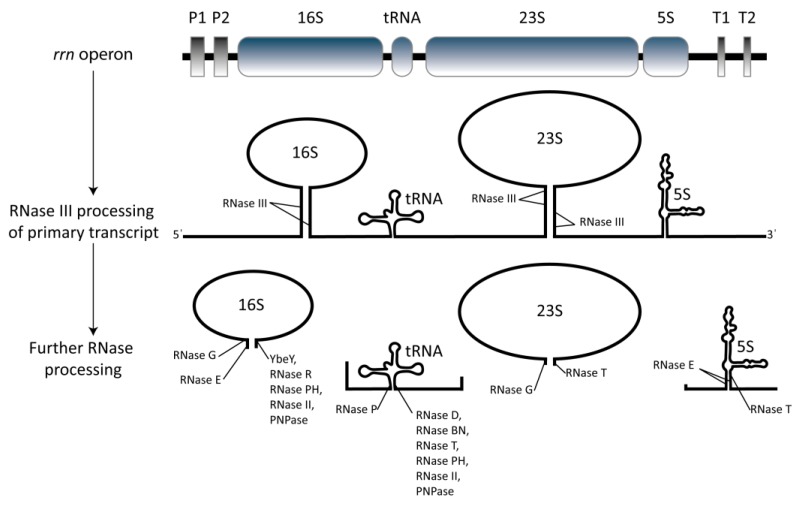
Schematic overview of rRNA production in prokaryotes using *E. coli* as an example. Production initiates with the transcription of a typical *rrn*-encoded rRNA operon by RNA polymerase into a single polycistronic transcript encoding 16S, tRNA, 23S and 5S (the presence of tRNA depends on the operon). Processing begins concurrently with transcription, with r-proteins and secondary rRNA structures beginning to assemble while chemical modifications occur. RNase III cleaves the primary transcript into the pre-16S (17S), pre-23S (25S), pre-tRNA and pre-5S through recognition of helical stem loops. Subsequent processing to generate mature rRNA is carried out by a range of ribonucleases as indicated, which differ depending on the organism. Enzymes involved in the 3′ processing of tRNA differ depending on the tRNA family and 3′ immature region in question [46].

While the assembly of r-proteins onto the immature subunits exhibits some modularity [2], the correct processing and protection of the rRNA is essential for the functionality of the ribosome. During the first step of the ribosome assembly pathway, the three rRNAs are cotranscribed into one precursor transcript from *rrn* operons (Figure 1). Different bacterial species contain differing numbers of *rrn* operons, which comprise up to 1% of the genome. For instance, *E. coli* contains seven operons, while *Bacillus subtilis* contains 10. Once transcribed, the precursor rRNA is subject to ribonuclease (RNase) III processing into the 17S, 25S, 5S and tRNA (Figure 1) [41]. Subsequent secondary structure formation of the immature 17S rRNA recruits associated ribosomal proteins during 30S formation, with several hierarchies of proteins present (reviewed by [38]). The primary r-proteins bind directly to the rRNA, secondary protein binding is reliant on primary protein presence and, lastly, tertiary protein binding to the secondary r-proteins occurs [1], with each binding event stabilising the rRNA fold [40]. 17S processing in *E. coli* is thought to occur on the ribosome itself, with rapid removal of 115 nucleotides from the 5′ by RNases E and G and 33 nucleotides from the 3′ via the activity of several RNases including the endoribonuclease YbeY and the redundant 3′–5′ exoribonucleases RNase R, PH, II and polynucleotide phosphorylase (Figure 1) [42,43,44]. The ribonucleases involved in rRNA maturation differ drastically between species, especially when comparing Proteobacteria and Firmicutes. Recent data has revealed that the fidelity of translation is somewhat governed by the accuracy of the 16S rRNA folding during 30S assembly, possibly through folding of 16S helix 44 (h44) [45], and as such the correct assembly of ribosomal proteins is crucial to the functionality of the ribosome and of every protein translated in the cell. The current model for 50S subunit assembly is similar to that of the 30S, in that the primary, secondary and tertiary proteins bind to the structured 5S and 23S rRNAs before self-assembly of the 50S takes place [38]. However, a complex network of interactions and the modular nature of 50S assembly have led to a better understanding of 30S assembly.

## 3. Domain Structure of RA-GTPases

**Figure 2:**
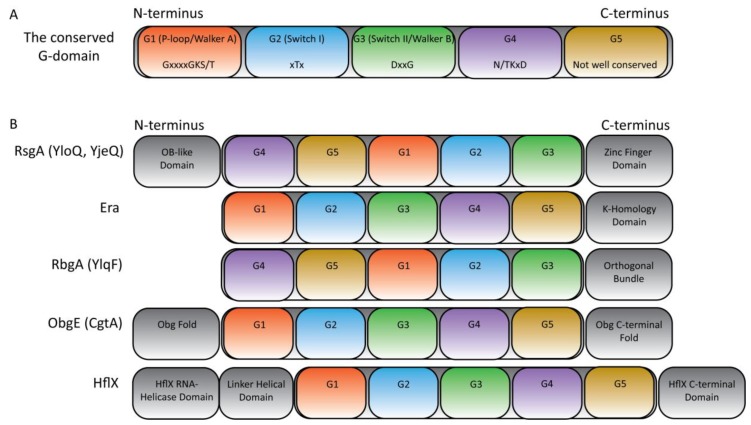
Domain structure of RA-GTPases inhibited by (p)ppGpp. (**A**) The general domain structure of the highly conserved GTPase domain, featuring the catalytic G1-G5 motifs is shown. Functions for these motifs include: G1 (P-loop, Walker A motif) binds and recognises the α- and β- phosphate of GTP; G2 and G3 (Switch I and Switch II/Walker B motif, respectively) stabilise the γ-phosphate of GTP through coordination of the Mg^2+^ cofactor; G4 confers guanine specificity via π-stacking of the lysine residue with the guanine ring and specific H-bonding of the aspartate with the guanine amino groups; G5 interacts with the guanine via H-bonds, but is poorly conserved and not universally present among TRAFAC GTPases. Consensus sequences are highlighted. (**B**) The domain architecture of the TRAFAC GTPases mentioned in this review. Note the circular permutation of RsgA and RbgA.

All P-loop GTPases are characterised by the signature central GTPase (G) domain, consisting of either four or five highly conserved G-motifs (G1–G5: Figure 2) that confer binding specificity and functionality to the active site [4,47]. G1, also known as the Walker A motif or P-loop, is present in the majority of identified nucleotide triphosphate (NTP) binding proteins (whether GTP or ATP binding). The consensus sequence GxxxxGKT/S enables the orientation of the α and β phosphate of NTPs to allow nucleophilic attack of the tertiary phosphate via the primary amino group of the conserved lysine [48]. The G2 motif, also known as switch I, is characterised by a conserved threonine in TRAFAC GTPases. This threonine residue coordinates the essential Mg^2+^ cofactor responsible for stabilising the γ-phosphate of the bound NTP, while also activating a water molecule in preparation for the hydrolytic S_N_2 nucleophilic attack [49]. The adjacent G3 motif, otherwise known as the Walker B motif or switch II, carries out a similar function to G2 and has a consensus of DxxG. During GTP hydrolysis, the Switch I and II regions undergo large conformational changes, which are thought to contribute to the molecular ‘switching’ behaviour of GTPases by transducing a signal to the accessory domains to alter activity or binding specificity [5]. G4 is characterized by the presence of four bulky hydrophobic amino acids followed by (N/T)(K/Q)xD, within which the conserved lysine/glutamine stack specifically against guanine nucleotide bases, while the aspartic acid residue forms specific bifurcated hydrogen bonds with the guanine secondary amines to act as a selectivity barrier against ATP [50]. The G5 motif interacts with the guanine base via water-mediated hydrogen bonds and is not strictly ubiquitous.

## 4. (p)ppGpp-Binding RA-GTPases Involved in 30S Assembly

### 4.1. RsgA (YloQ/YjeQ)

The multidomain GTPase RsgA (YloQ in *B. subtilis*, YjeQ in *E. coli*) is a late-stage 30S ribosome assembly cofactor that is widely conserved among bacteria, although is not essential for growth [6,51]. It contains the conserved G-motifs arranged in an alternative G4-G5-G1-G2-G3 permutation (Figure 2), making it a member of the circularly-permuted GTPase family (CP-GTPase) alongside the other ribosome biogenesis cofactors YawG, YqeH and RbgA. The switch II region is, thus, located towards the C-terminus of the protein in an unstructured state [52]. Consequently, an additional domain is required for stabilisation that can either be independent or dependent on cofactor binding, and indeed RsgA has an N-terminal OB (oligonucleotide/oligosaccharide-binding) domain and a C-terminal zinc-finger domain, both of which are implicated in binding to RNA (Figure 2) [53]. Despite the availability of the structure of RsgA in multiple nucleotide-bound configurations by both X-ray crystallography and cryo-electron microscopy (cryo-EM) [53,54,55], the mechanism of GTP catalysis has not been elucidated due to the instability of the switch I region during crystallography and the low resolution of cryo-EM. It has been proposed that RsgA uses a catalytic residue from the switch I region that is activated upon correct maturation of the h44 of the 16S rRNA [56].

**Figure 3:**
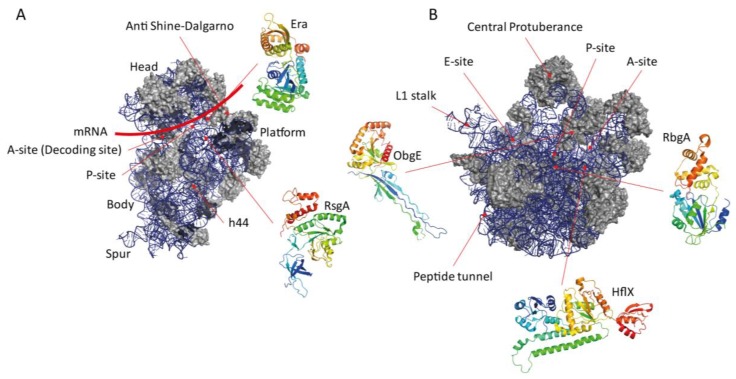
An overview of the ribosomal binding sites and structures of (p)ppGpp-binding RA-GTPases. (**A**) *E. coli* 30S ribosomal subunit (adapted from PDB-ID 4ADV [60]) with major domains and features labelled. The binding sites for RsgA and Era are indicated (structures adapted from PDB-IDs 5NO4 [56] and 3IEV [61], respectively). (**B**) *E. coli* 50S ribosomal subunit (adapted from PDB-ID 3J5L [62]) with major structural features including the A-, P- and E-sites labelled. The binding sites for ObgE, RbgA (not present in *E. coli*) and HflX are indicated (structures adapted from PDB-IDs 5M04 [63], 3CNL [64] and 5ADY [65], respectively). R-proteins are coloured grey, whereas rRNA is coloured blue for comparison. GTPases are coloured as a gradient from the N-terminus (blue) to the C-terminus (red) and are not to scale relative to the ribosome.

RsgA binds the 30S subunit at the 30S–50S interface with high affinity (66.2 ± 7.7 nM) [57], with the G domain and N-terminal OB domain binding the 30S body and the C-terminal zinc-finger contacting the 30S head (Figure 3) [56,58]. Binding of RsgA to the immature 30S subunit is associated with the docking of h44 of the 16S rRNA in the correct conformation, leading to the hypothesis that RsgA monitors h44 to ensure correct positioning prior to 30S maturation [53,54,56]. Upon binding, the OB domain of RsgA is positioned within the A-site of the 30S subunit, with residues F48–H51 inserted into the minor groove of h44. The G domain clamps h44 in close proximity to h24 and h45, stabilising it through interactions with the OB domain and the switch I region of the G domain. The zinc-finger domain binds the 30S head at a position involved in interactions between the charged tRNA and the ribosomal P-site [56]. Taken together, the localisation of RsgA during 30S binding suggests that this protein can monitor the maturation state of the 30S tRNA and mRNA binding sites [56]. Interestingly, these three contact points only occur in the GTP-bound form of RsgA; in the GDP bound form the OB domain is the primary point of interaction. RsgA can also interact with mature 70S ribosomes, resulting in dissociation into the 30S and 50S subunits, while destabilising the r-proteins uS2, uS3, uS7, uS12 and bS21 [56,59]. The implications of this being that RsgA can rescue kinetically trapped intermediates by encouraging release of r-proteins that have associated incorrectly.

A non-GTPase assembly cofactor for the 30S, RbfA (ribosome binding factor A), binds to the 5′ end of the 16S rRNA, possibly during the 17S immature stage, to enable destabilisation during h1 development and, perhaps, RNase processing [66]. RbfA is a bacterial cold-shock protein, consisting of a type-II KH (K-homology) domain characteristic of nucleic acid binding proteins. As well as facilitating the formation of h1, RbfA binding is followed by dramatic alterations in h44 and h45 conformations to prohibit subunit joining [67]. The association of RsgA with the 30S can release RbfA when in the GTP-bound state, enabling RsgA-mediated positioning of h44 and h45 into the correct conformation [66]. This is consistent with previous studies showing that RsgA is unable to stably bind the 30S in the absence of GTP [59,68]. Since release of RbfA was also observed when incubated with the RsgA in the presence of a non-hydrolysable GTP analogue GMPPNP, it is apparent that binding of RsgA to the 30S is enough to promote RbfA release and could be involved in controlling RbfA-mediated maturation events [66].

### 4.2. Era

Era is a highly conserved protein across prokaryotes and eukaryotes with pleiotropic roles within the cell, including ribosome assembly, cell cycle control and apoptosis [69,70,71]. Era is a two-domain protein featuring a C-terminal KH RNA-binding domain as well as the N-terminal G domain (Figure 2). The KH domain is responsible for binding to the 3′ end of the 16S rRNA adjacent to the anti-Shine-Dalgarno (SD) sequence (Figure 3) [72]. Here it recognises the GAUCA motif, which along with h45 (nucleotides 1506-1529) is universally conserved [73]. The crystal structure of Era bound to GMPPNP exhibits a closed domain structure in which the G and KH domains close around the ligand, whereas in the apo and GDP-bound states the domain structure is open [61]. This displacement covers a range of approximately 10–15 Å and may suggest that Era binding to the 30S depends on the bound nucleotide. In the mature 30S this binding site is occupied by bS1—the final r-protein in the 30S assembly pathway; Era and bS1 from *E. coli* cannot coexist on the pre-30S particle [59]. While the GAUCA motif is crucial for Era binding to the 16S, optimum GTPase stimulation is reliant on the adjacent anti-SD sequence (CCUCC), so altogether the correctly matured _1530_GAUCACCUCC_1539_ region is required for up to 12-fold stimulation of Era activity [73,74].

It has been determined experimentally that Era binding to the ribosomal subunit interface can prevent 30S and 50S joining, indicating that Era is important as a checkpoint for 16S maturity prior to 70S assembly [75]. Indeed, deleting *era* in *Staphylococcus aureus* caused a marked decrease in 70S formation, with an increase in mature 50S subunits relative to 30S, indicating a 30S processing defect [76]. Cryo-EM data from strains with controlled *era* depletion in *E. coli* accumulate 30S subunit assembly intermediates ranging from late-stage to early-stage, and also reveal that the 30S platform region remains unfolded in the absence of Era, with densities lacking for uS2, uS5 and bS21 [77]. Similarly, the density maps of h23 and h24 are highly fragmented and h44 and h45 were completely absent [77]. The authors thus suggest that the folding of h23 and h24 is a major kinetic barrier in the correct maturation of the 30S subunit, especially the head region, and that Era facilitates the efficacy of this process [77].

It has been shown that, during 30S pre-initiation complex (30S pre-IC) formation, IF3 binds to the 16S rRNA at nucleotides 1532–1534 within the GAUCA motif [78]. The 30S pre-IC is a complex of IF1, IF2, IF3, mRNA and N-formylmethionine (fMet)-tRNA, which assembles prior to 70S formation and translational initiation and is an absolute requirement for 30S-50S joining into a translating 70S particle [79]. Mutation of _1530_GA_1531_ also affects IF3 association via alteration of the 16S 3′ conformation, which differs from the Era-bound and IF3-bound states [80]. Due to the overlap of Era and IF3 binding sites, it appears that Era occludes IF3 binding and 30S pre-IC formation while bound to the 16S. Taken together with the fact that Era binding also occludes the anti-SD sequence and, thus, prevents mRNA recruitment to the 30S pre-IC, it is feasible that Era-mediated 16S maturation is in actuality the final stage of 30S assembly before the initiation of translation.

A number of studies using mutant bacterial strains also shed light on the cellular function of Era. This protein is essential for growth in *E. coli*, although can be deleted in *S. aureus* [76,81,82,83]. Overexpression of Era can partially suppress the phenotypes present in Δ*rsgA* mutants, indicating an overlap of function [54,66,76]. Era may however, function downstream of RsgA, given that a 20-fold excess of Era increases the dissociation constant of RsgA binding to the 30S from 58.5 nM to 2.3 μM through destabilisation of h44 [77]. 16S rRNA processing remains a crucial aspect of 30S assembly and deletion of the highly conserved, multifunctional endoribonuclease YbeY (Figure 1) in *E. coli* imparts a striking defect in 16S processing at both the 3′ and 5′ ends [84]. Δ*ybeY* strains of *E. coli* accumulate 17S and truncated 16S rRNA (16S*) lacking the 3′ terminus [85]. While mature 16S rRNA is present within these cells, the excess of immature rRNA leads to the production of defective 30S subunits resulting in defects in 70S ribosome profile [85]. The *ybeXYZ* and *era* genes are located within the same operon in many bacteria, indicating a functional relationship, and indeed YbeY has been shown to interact directly with Era and the r-protein uS11 adjacent to the Era binding site [86,87]. The YbeY-Era interaction is mediated by the G domain of Era, while the opposite side of the YbeY endonuclease domain contacts uS11 directly. This has led to the proposition of a model where Era is responsible for recruiting YbeY to the immature 16S rRNA to undergo processing [76,85]. However, overexpression of Era in Δ*ybeY* strains improves the 16S processing defect, 70S assembly and partially rescues growth in a GTPase-dependent manner [86]. This indicates that other exoribonucleases such as RNase R, RNase PH and RNase II that are likely involved in 16S processing can compensate for a deletion of *ybeY*, however, the mechanism behind this is not clear [86]. Evidence for Era functioning as a hub protein also come from the fact that Era can interact with the DEAD-box RNA helicase CshA in *S. aureus* [76], which had previously only been shown to be involved in 50S biogenesis through direct subunit binding [88]. A deletion of *cshA* in *S. aureus* showed a defect in 16S maturation at 25 °C, supporting the suggestion that Era acts as a scaffold protein for enzymes involved in 16S rRNA maturation and processing [76]. However, evidence for CshA directly processing 16S rRNA is lacking, meaning that indirect effects cannot be discounted.

## 5. (p)ppGpp-Binding RA-GTPases Involved in 50S Assembly

### 5.1. ObgE (CgtA)

ObgE (CgtA in *B. subtilis*) is an essential GTPase that is highly conserved amongst prokaryotic and eukaryotic organisms. Similar to Era, ObgE is thought to be involved in a plethora of cellular functions including ribosome assembly, modulation of the stress response, sporulation, persistence, cell division, chromosome segregation and DNA replication [63,89,90,91,92,93]. The conserved, three domain structure of the Obg family consists of a diverse range of C-terminal domains that are implicated in RNA binding and dimerisation, a central G domain and a highly conserved N-terminal domain that is a structural analogue of tRNA (Figure 2) [93].

Co-sedimentation assays have revealed that ObgE associates with the 30S subunit weakly and the 50S subunit strongly in the presence of GTP, GMPPNP and the stringent response alarmone ppGpp, with ppGpp increasing the occupancy of the 50S five-fold [93]. Consistently, ObgE has been shown to co-sediment with both the 16S and 23S rRNA in a homodimeric and GTP-dependent manner [94]. Pull-down assays have identified a number of proteins with which ObgE associates, including uS3, uS4, uS5, uS13, bS16, uL2, uL4, uL16, bL17, RNAP β-subunit, the stringent response synthetase/hydrolase SpoT and the RNA helicase CsdA [94]. The N-terminal domain of ObgE is a structural mimic of the tRNA A-site acceptor arm and indeed overlaying the 50S:ObgE and P-site tRNA structures reveals that residues 29-31 of the N-terminal domain of ObgE is capable of contacting the CCA motif of the P-site tRNA [93]. K31 inserts between A76 and S2451 of the tRNA and 23S rRNA, respectively, in a similar way as release factor 2 (RF2), which recognises the termination codon on the template mRNA [93,95]. Furthermore, the location of ObgE on the 50S (Figure 3) is in close proximity to the methylation sites targeted by RrmJ, RluD and RluC [93], the modification of which is instrumental to the functionality of the 23S rRNA. This methylation is also thought to enhance the GTPase activity of ObgE 120-fold in the presence of the mature 50S subunit [93]. ObgE binding to the 50S also prevented association of the 30S and 30S pre-IC in nucleotide-bound forms. Interestingly, comparison between the IC_50_ of ObgE involved in prevention of subunit joining showed that the inhibitory effect is much stronger in the case of the 30S pre-IC than the naked 30S subunit [93], implicating ObgE as a very late-stage assembly cofactor. Taken together, the position of ObgE on the 50S has been interpreted to prevent premature association of tRNAs to the A-site via steric occlusion, as well as monitoring the methylation state of the 23S rRNA prior to GTP hydrolysis and dissociation to enable the initiation of translation. ObgE has also been shown to be capable of 70S splitting in a nucleotide-independent manner when in at least 30-fold excess [93]. However, due to the excess of ObgE used in this experiment, the physiological relevance of this is questionable.

While ObgE is essential, temperature-sensitive mutants have been produced and used to study the effect of ObgE mutation on rRNA processing in the cell. These mutants form immature dead-end 50S* particles lacking uL16, bL33 and bL34. In ObgE-depleted cells, 16S rRNA processing is severely impaired via prevention of 17S 3′ and 5′ cleavage and, as a result, the 70S ribosome content of the cell decreases [94]. The interaction between ObgE and CsdA has led to the hypothesis that ObgE recruits CsdA to the immature 16S or 23S rRNA, which is then unwound by the helicase activity of CsdA prior to processing. ObgE is also thought to function as a relatively late stage 50S assembly factor, as it exhibits a lack of association to the 40S intermediates formed in RNA helicase mutant Δ*csdA* and Δ*srmB* strains, suggesting it binds after these processing events [96].

### 5.2. RbgA (YlqF)

Another late stage 50S assembly cofactor, RbgA (YlqF in *B. subtilis*), is an essential protein in the Firmicutes but is completely absent from Proteobacteria. RbgA displays a two-domain architecture: a circularly permuted N-terminal G domain, which contains an unusual K-loop, and a C-terminal helical ANTAR domain (AmiR and NasR transcription anti-termination regulator) implicated in RNA binding and potential rRNA remodelling (Figure 2) [64,97]. Interactions between the C-terminal domain and the 23S rRNA leads to protection of nucleotides C928, C942, A2301 and A2354 [98], which are located in helices 38, 38, 81 and 85, respectively. H81 and 85 are positioned in close proximity to the uL5 protein responsible for tRNA interaction within the P-site, so dissociation of RbgA following the stimulation of GTPase activity by the correctly conformed 23S rRNA may enable uL5 docking. Mutation of the ANTAR domain has been shown to negatively impact the GTPase activity of RbgA by preventing 50S interaction. The circular permutation of the G domain is thought to transduce conformational changes to the C-terminal domain, which can influence rRNA binding and, thus, ribosome association in complex with different nucleotides [97].

Depletion of RbgA in *B. subtilis* results in the formation of immature large ribosomal subunits which migrate at a density of 45S, however, it is currently unknown whether these intermediates are capable of maturing into the active 50S, or whether depletion of RbgA results in a dead-end non-functional complex [99]. Furthermore, this 45S intermediate is lacking uL16, bL27 and bL36—three r-proteins that contribute to the integrity of the A- and P-site of the 50S subunit, and are postulated to be involved in both A- and P-site tRNA binding [98,100]. uL16 interacts directly with h38, whereas bL27 interacts with h81, further implicating RbgA in cofactor recruitment following correct 23S maturity. In the presence of GMPPNP, RbgA can stably interact with both the 50S and 45S particles (Figure 3), however, when bound to GTP, interactions with the 50S are transient due to 60-fold GTPase stimulation upon binding—indicating a GTP-dependent binding mechanism [99].

Mutation of amino acid F6 to A in RbgA reduces GTPase stimulation by the 50S subunit 12-fold, and was used to carry out suppressor screening [100]. It was observed that all suppressor mutations mapped to the *rplF* gene encoding the r-protein uL6, with six unique mutations generated: R3C, G5C, G5S, H66L, T68R and R70P – revealing a groove on L6 which appears to be responsible for suppressing the growth defect of RbgA-F6A. Interestingly, the suppressor strains accumulated novel 44S intermediates, distinct from the 45S intermediates isolated from *rbgA* mutant strains [100], as well as exhibited an increase in 70S ribosomes as compared to RbgA-F6A. These 44S particles are able to mature in vitro into functional 50S particles, which, despite exhibiting a defect in subunit joining, might explain the suppression of the growth defect present in RbgA-F6A mutants [100]. Despite the absence of direct association between RbgA and uL6, the suppressor mutations are thought to destabilise the uL6-50S interaction, increasing the probability of uL6 association with the correctly oriented 23S rRNA. The role of RbgA may be to properly position the uL6 interaction with h97 and with the sarcin-ricin loop, which then enables tertiary r-proteins uL16, bL27, bL28, bL33, bL35 and bL36 to incorporate. This incorporation could then induce the catalytic step associated with GTP hydrolysis and dissociation of RbgA [100].

### 5.3. HflX

Previously characterised as both an ATPase and GTPase, the universally conserved HflX has been implicated in manganese homeostasis, ribosome binding, 70S splitting and even RNA secondary structure unwinding [65,101,102,103,104,105]. Two isoforms of HflX exist in nature, the first is found in organisms such as *E. coli* and *S. aureus* and consists of a three domain architecture—the conserved N-terminal HflX domain, the central G domain and the C-terminal domain of unknown function (Figure 2) [104]. The second, atypical isoform lacks the C-terminal domain and has been found in the hyperthermophilic archaeon *Sulfolobus solfataricus* [106]. A helical linker region connects the N-terminal domain and G domain. Interestingly, the HflX N-terminal domain contains a unique nucleotide binding fold, capable of binding ATP without the use of a canonical Walker A nucleotide binding motif [107]. Several years later this domain was shown to exhibit ATP-dependent RNA helicase activity, for which the helical linker is crucial [104]. This linker domain interacts with domain V of the 23S rRNA following a dramatic, 20 Å conformational change in response to ATP binding, which is a prerequisite for rRNA unwinding [104]. The ATPase activity is stimulated by an aberrant form of the 50S, generated in vitro by heat shock and 1.5 M NaCl washing. As such, it is logical to assume that one role of HflX is to repair incorrectly folded rRNA during the assembly process [104]. It has also been demonstrated that HflX is a heat-shock protein encoded in an operon downstream of the gene encoding the universal stress response protein Hfq, which is under the control of a heat sensitive promoter in *E. coli* [108,109,110]. Cells lacking Hfq contain higher levels of immature 30S ribosomes compared to the wild type, suggesting the general stress response is linked to 30S assembly via this protein [111,112]. This may imply that HflX could have a role in the repair of abnormal 50S subunits generated during conditions of heat stress.

Contradictory reports have shown that HflX can bind to the 30S, 50S and 70S ribosomal particles in the presence or absence of guanosine nucleotides, with ribosome binding enhancing the stability of the nucleotide-bound state [103,106,113]. To clarify the issue, Zhang et al. carried out isopycnic ultracentrifugation of HflX with each subunit in the presence of different nucleotides [65], revealing an enrichment of 50S association when bound to GMPPNP, with dissociation requiring GTP hydrolysis. A similar nucleotide-dependence was observed for 70S binding, accompanied by a strong splitting activity of both the isolated 70S particle and puromycin-treated polysomes, with GMPPNP-bound HflX splitting 10-fold more efficiently than the apo GTPase [65]. Notably, the presence of peptidyl-tRNA in the ribosomal P-site, close to the HflX binding site, partially inhibited GTPase activity [65,110]. This suggests the preferred substrate of HflX splitting is translationally stalled 70S, containing a de-acylated tRNA in the P-site that is characteristic of translationally-stalled ribosomes. Subunit recycling by HflX could be involved in the rescue of these stalled ribosomes. Cryo-EM structures reveal that the binding site of HflX is in fact the subunit interface (Figure 3), with the N-terminal helicase domain protruding towards the PTC and contacting several rRNA helices. This provides a hypothesis as to why GTP hydrolysis is not required for 70S splitting, as HflX binding disrupts bridge B2a between h69 and the 16S rRNA towards the 3′ of h44 [114] in a similar manner to RRF (Ribosome Recycling Factor) during standard post-translational recycling [65]. It is, therefore, apparent that HflX can act as both an anti-association factor, inhibiting 70S formation until GTP hydrolysis removes HflX from the 50S, and as a splitting factor, rescuing stalled ribosomes under stress [65].

During conditions of relatively low nutritional availability, bacteria form 100S ribosomal complexes consisting of two 70S particles stabilised by HPF (Hibernation Promoting Factor) in Firmicutes, and HPF in conjunction with RMF (Ribosome Modulation Factor) in γ-Proteobacteria [115]. The former arranges in a side-to-side configuration, whereas the latter arranges in a head-to head configuration. In Firmicutes, 100S complexes are produced constitutively throughout exponential and stationary phase, whereas Proteobacteria and Cyanobacteria only produce the 100S complexes during stationary phase and, in the latter case, darkness [116,117]. HPF occludes the PTC of the 50S ribosomal subunits, rendering the 100S translationally inactive. While IF3, EF-G and RRF have been implicated in 100S dissociation *in vivo*, the predominant splitting factor is yet to be identified. It has, however, been shown that HflX can split the 100S ribosome into the 70S constituents in a GTPase-dependent manner, as HflX-GTP showed strong splitting activity in stark contrast to HflX-GMPPNP, which was incapable of splitting the 100S [115]. It is unlikely that HflX is the unidentified primary 100S splitting factor, as it is mainly transcribed during heat shock conditions and has a much lower level of transcription during exponential growth [108].

## 6. Interplay between RA-GTPase Activity and the Stringent Response

**Figure 4:**
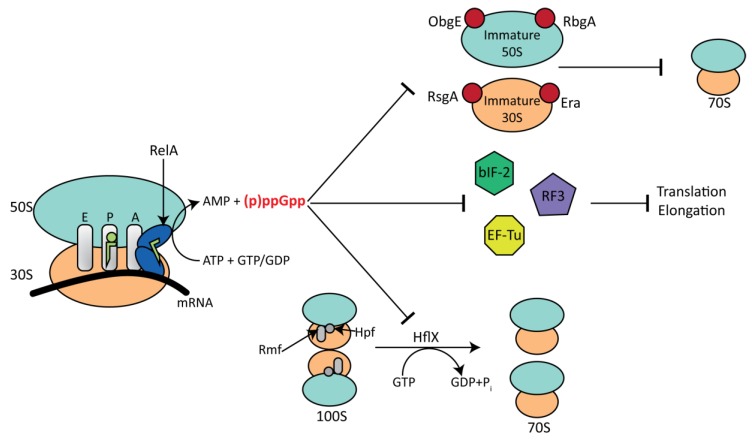
The effect of the stringent response on ribosome maturation and translation. During the stringent response the alarmones (p)ppGpp accumulate following synthesis by RSH-superfamily enzymes. In *E. coli,* the interaction between RelA (dark blue) with the ribosome activating complex (RAC) increases the synthesis of (p)ppGpp. The RAC consists of the ribosome (50S, lightblue; 30S, orange), mRNA and uncharged tRNA (green). A charged tRNA is situated in the P site of the ribosome. (p)ppGpp inhibits the GTPase activity of various small GTPases, preventing correct ribosome maturation. (p)ppGpp also inhibits the splitting of inactive 100S ribosome dimers by HflX but promotes splitting of 70S ribosomes to inactive 50S and 30S subunits. Furthermore, (p)ppGpp inhibits the translational GTPase bIF-2, EF-Tu and RF3, resulting in a reduction in translation.

Recent genome-wide screening has revealed that (p)ppGpp can bind to, and inhibit the GTPase activity of, the RA-GTPases RsgA, RbgA, Era, HflX and ObgE in both Gram-negative and Gram-positive bacteria (Figure 4) [6,118], leading to a reduction in the formation of the 70S ribosome and attenuated growth. ppGpp has a higher affinity for these GTPases than GTP, whereas pppGpp and GTP bind with similar strengths [6]. Interestingly, the RsgA homologue in *B. subtilis* was unable to bind (p)ppGpp [6], potentially due to amino acid differences in the (p)ppGpp binding site. The mechanism of GTPase inhibition is currently uncertain, however, crystal structures of RbgA, the *E. coli* GTPase BipA and the DNA primase DnaG have been solved in the (p)ppGpp-bound states, revealing that (p)ppGpp acts as a competitive inhibitor via binding to the G domain in a near identical manner to GTP [119,120,121,122]. Binding of ppGpp to the nucleotide-binding pocket of BipA had no effect on the tertiary structure of the enzyme, suggesting that inhibition is purely due to outcompeting GTP binding [120]. Inhibition of DnaG has been studied in much greater detail. The (p)ppGpp binding site overlaps that of the priming RNA NTPs, with the 5′ phosphate tail binding in an identical manner [122]. However, the presence of the 3′ diphosphate in the alarmones enables direct interaction with an associated Mn^2+^ cofactor, which consequentially results in a reconfiguration of the guanosine base into an orientation distinct from that of the NTPs. Due to this, it was concluded that inhibition of DNA primase relies on blocking entry of incoming NTPs to the binding site through use of the innate nucleotidyl properties of (p)ppGpp [122]. As a result of the specificity of the binding site of RA-GTPases to guanine rings via stacking of a lysine residue and specific bifurcated hydrogen bonding between an aspartate carboxyl and substrate nitrogen atoms [123], it can be speculated that (p)ppGpp inhibition of these enzymes may take place in a similar manner to that of DnaG. Indeed, the crystal structure of RbgA reveals (p)ppGpp bound in the GTP-binding pocket, however, the position of the switch I (G2) loop differs when compared to the GMPPNP bound form of Lsg1, an RbgA homologue from *Saccharomyces cerevisiae*, with incorrect loop docking taking place in the former. This led to postulation that the δ and ε phosphate moieties of (p)ppGpp sterically prevent the switch I loop from associating correctly, preventing hydrolysis of (p)ppGpp and prolonging the inhibitory effect [119]. The decrease in GTPase activity of RbgA in the presence of ppGpp [6,119] may lead to a decrease in RbgA release and, thus, the sequestering of immature 45S and mature 50S ribosomal intermediates.

In addition to (p)ppGpp binding, the GTPase Era has been shown to directly associate with the bifunctional (p)ppGpp synthetase/hydrolase Rel*_Sau_* in *S. aureus* via the conserved G domain [76]. This interaction increases the GTPase activity of Era, perhaps increasing the dissociation of Era from the 30S during stressed conditions to regulate ribosome assembly. Both Era and Rel*_Sau_* also interact with CshA, a DEAD-box RNA helicase, potentially as part of an Era-CshA-Rel*_Sau_* complex. In the presence of Rel*_Sau_*, the helicase activity of CshA was inhibited, thus limiting the impact of this enzyme on ribosomal biogenesis [76].

Despite the likely similarity in (p)ppGpp binding mechanics between HflX and ObgE and the previously discussed RsgA, RbgA and Era, the resulting effects are very different. Under normal, GTP-bound conditions, HflX functions as a 100S and 70S splitting factor that can also prevent 30S and 50S subunit joining [115]. In the ppGpp-bound state, HflX is able to split the 70S ribosome with comparable activity as the GMPPNP-bound state, and can bind to, but remains unable to dissociate from, the 100S hibernating complex due to the lack of GTP hydrolysis, preventing splitting into functional 70S ribosomes [115]. In *S. aureus*, growth at 47 °C stimulates the activity of HflX, yet Δ*hflX* strains are more viable at this higher temperature than the wild type, most likely due to an observed increase in translational efficiency in the knockout. This induction of *hflX* was alleviated in a Δ*sigB* mutant, with a twofold reduction in expression [115]. A Δ*rel_Sau_* strain of *S. aureus* derepresses the expression of both HPF and HflX through an unknown but (p)ppGpp-mediated mechanism [115]. This implicates an accumulation of (p)ppGpp within the cell with increased expression of HflX, which encourages splitting of the active 70S into inactive 30S and 50S particles. These can then be sequestered by other RA-GTPases in the (p)ppGpp-bound state to arrest translation during low-energy or stressed conditions.

When associated with GTP, ObgE is a 50S maturation factor capable of associating with a range of r-proteins, playing a vital role in PTC development as well as other cellular events, such as DNA replication. During nutrient-rich conditions, the ObgE homologue CgtA from *Vibrio cholera* can bind to and repress the synthetase activity of the long RSH enzyme SpoT [24]. However, during stringent response activation, ObgE can bind to ppGpp with a similar affinity to GDP as part of a positive feedback mechanism [93]. Co-sedimentation assays showed greater association of ObgE to the 50S when bound to ppGpp rather than GTP or GMPPNP [93], corroborating the role of ObgE as an anti-association factor. Shifting the 70S dissociation equilibrium towards the constituent subunits during the stringent response may act as a further mechanism by which translation is stalled and growth is arrested, but may also enable greater subunit degradation, a process which begins via 70S splitting [124]. Interestingly, a growth-suppressed but viable *E. coli obgE*::Tn5 insertion strain had a ppGpp:pppGpp ratio much lower than seen in the wild type [90], which can be completely reversed by overexpression of native ObgE. ppGpp has been named the major controlling factor that influences growth rate in *E. coli*, suggesting that ObgE has an important role in facilitating the efficacy of the stringent response via conversion of pppGpp to ppGpp. This process is usually carried out by the GppA 5′ γ-phosphatase, but can also be achieved by the hydrolase SpoT, which has been shown to interact with ObgE in vivo [94,125]. Studies have shown that a lower ppGpp:pppGpp ratio has a weaker inhibitory effect on cellular RNA synthesis, potentially due to a reduction in RNAP control by ppGpp [125], which also results in a greater growth rate due to continued transcription of rRNA. From this it seems that ObgE enhances the conversion of pppGpp to ppGpp via an unknown mechanism, which facilitates the stringent response-mediated effects of many cellular components including the RA-GTPases mentioned here.

Overall, it appears that the stringent response can interact with ribosome assembly GTPases either via the stringent response alarmone nucleotide (p)ppGpp to inhibit the GTPase activity of these RA-GTPases and, thus, alter their cellular functions, or through direct protein-protein interactions between the (p)ppGpp synthetase/hydrolase enzymes and specific RA-GTPases [76,90]. The stringent response then reduces the cellular pool of 70S ribosomes in order to reduce the growth rate and facilitate degradation under conditions of stress through either a reduction in subunit maturation rate or an increase in mature 70S splitting.

## 7. Inhibition of rRNA Transcription and Processing by the Stringent Response

**Figure 5:**
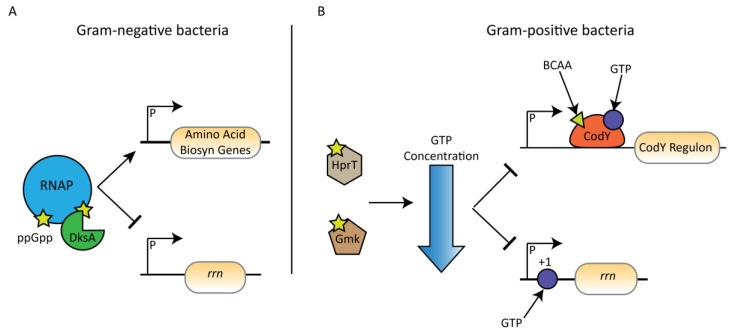
The effect of the stringent response on transcription in Gram-negative and Gram-positive bacteria. (**A**) In Gram-negative organisms, (p)ppGpp (yellow) and DksA (green) bind to RNA polymerase to alter the transcription of amino acid biosynthesis genes (activated) and rRNA genes (repressed). (**B**) In Gram-positive bacteria (p)ppGpp bind to and inhibit multiple enzymes involved in GTP synthesis. This coupled with the decrease in branched chain amino acid (BCAA) levels during the stringent response results in a derepression of the CodY regulon as well as a decrease in transcription of *rrn* and other genes with a G residue at the +1 site.

The stringent response result in a very large change in transcriptional profile in *E. coli*, with over 700 genes differentially expressed, including a decrease in rRNA synthesis and a concurrent increase in expression of amino acid biosynthesis and transport genes [10,126,127]. In *E. coli*, (p)ppGpp can bind directly to two sites on the RNAP complex, although ppGpp is the more potent effector (Figure 5) [125,128]. Site 1 comprises a cleft surrounded by the β’ and ω subunits of the RNAP, with binding site mutations eliminating the inhibition of transcription in the presence of (p)ppGpp [125,128,129]. The ability of an RNAP from a given species to bind to (p)ppGpp at this site can be predicted by the presence of a methionine-alanine-arginine (MAR) motif at the N terminal end of the ω-subunit. This MAR motif is conserved amongst Alpha-, Beta-, Gamma- and Deltaproteobacteria but is absent in other classes, such as Firmicutes [16]. The effect of (p)ppGpp on reducing rRNA gene transcription is potentiated by binding of the alarmones to site 2, which is created when the transcription factor DksA binds to the β’ subunit of the RNAP [129,130]. DksA and ppGpp act synergistically, with the effect on RNAP transcription in the presence of both factors much greater than with either one alone. Indeed, both factors are required for the positive activation of amino acid biosynthesis gene promoters during the stringent response [131], as well as the inhibition of transcription from the P1 promoters of all 7 *rrn* genes in *E. coli* [132]. This is thought to occur via a destabilisation of the short-lived open complexes that form at *rrn* gene promoters [130,133]. (p)ppGpp appears also to regulate transcription even in non-stressed conditions. In *E. coli*, low ppGpp levels trigger the activation of the leucine response transcriptional regulator Lrp, whereas high ppGpp levels are required to activate the stress-response RpoS regulon [134].

In Gram-positive bacteria (p)ppGpp does not bind to RNAP and the cofactor DksA is not present. ppGpp has no effect on the stability of the DNA-RNAP open complex in *B. subtilis* and so cannot affect transcription through the same mechanism as in *E. coli* [135,136]. Instead, transcription is controlled through GTP levels, which are lower during the stringent response (Figure 5). GTP is used as a substrate in the production of (p)ppGpp, which then acts to directly inhibit GTP biosynthesis. Three enzymes in the GTP biosynthesis pathway are inhibited by (p)ppGpp: the IMP dehydrogenase GuaB; the hypoxanthine phosphoribosyltransferase HprT; and the guanylate kinase Gmk [136,137,138]. This lowered GTP pool contributes to a reduction in transcription by two mechanisms. Firstly, GTP is the initiating nucleotide of *rrn* promoters, so when GTP levels are low in the cell rRNA transcription is downregulated due to a slower initiation rate [135]. This method of rRNA regulation seems specific to Gram-positive bacteria, as the identity of the initiating nucleotide in *E. coli* has no effect on the promoter regulation by (p)ppGpp [139]. In *S. aureus*, G residues in positions +1–+4 can have a role in promoter activity and so any other gene with this property would also be downregulated during the stringent response [20].

In *B. subtilis*, incorrect maturation of either the 3′ or 5′ end of tRNA can induce the stringent response in a manner similar to RelA sensing the uncharged tRNA that accumulates during nutrient starvation [13]. This activation of the stringent response subsequently inhibits 3′ processing of the 16S RNA much more quickly than it does the inhibition of rRNA synthesis, preventing existing ribosomal precursors from maturing into functional 70S complexes [13]. Late-stage processing of the 16S rRNA is a crucial aspect of quality control, preventing subunit degradation by RNase R upon correct maturation [140], hence the stringent response not only shuts down de novo production of the ribosome constituents but also encourages the degradation of extant ribosomal precursors upon induction.

Secondly, GTP is one of two CodY cofactors, along with branched chain amino acids (isoleucine, leucine and valine) [141]. CodY is a gene repressor in Gram-positive bacteria with a large regulon [142]. As the GTP pool in the cell drops during the stringent response, the repression of CodY is released resulting in a very large change in the transcriptional profile. In *S. aureus*, 150 genes are up-regulated during the stringent response, however, only seven of these do so independently of CodY derepression [143]. A total of 161 genes are down-regulated during the stringent response induced by leucine/valine starvation and all of these are regulated independently of CodY. This is perhaps unsurprising seeing as how CodY generally acts as a gene repressor. This clearly highlights that, in Gram-positive bacteria, CodY is an important factor for determining gene up-regulation during the stringent response but not down-regulation.

## 8. Inhibition of Translation by the Stringent Response

In addition to controlling the transcription of rRNA and ribosome subunit assembly, (p)ppGpp also inhibits translational GTPases that are involved in the sequential process of protein production, namely bacterial initiation factor 2 (bIF-2), elongation factor Tu (EF-Tu) and recycling factor 3 (RF3) [118,144,145,146]. Once the mature 30S subunit is assembled, mRNA can bind to bS1 and, thus, associate with the 30S subunit. bIF-2 is recruited, which enables association of the initiator fMet-tRNA^Met^ to form Watson-Crick base pairing interactions between the mRNA start codon and the cognate tRNA anticodon. bIF-1 and bIF-3 facilitate bIF-2 and mRNA-30S binding, respectively [79], to form the 30S pre-IC. The 50S subunit then associates, with bIF-2 dissociating to remove the steric hindrance to 70S formation [147]. bIF-2 can bind to ppGpp in an identical fashion to GDP, maintaining the same hydrogen bonding networks [148]. It is well documented that bIF-2 can associate to the 30S pre-IC with high affinity in the GTP-bound state, but not while bound to GDP [145]. When interacting with (p)ppGpp, association of the fMet-tRNA^Met^ to the 30S pre-IC is negatively affected, with the rate of the initial dipeptide bond formation decreasing [148].

Following the formation of the 30S pre-IC and initiation of transcription, EF-Tu is responsible for escorting the aminoacyl-tRNAs to the A-site of the ribosome in preparation for residue incorporation [149]. Upon the correct situation of the tRNA, the GTPase activity of EF-Tu is activated and release from the A-site occurs. Subsequent association of EF-G close to the A-site provides energy to translocate mRNA and tRNA through the canonical and hybrid states while maintaining frame. Furthermore, recent structural studies have revealed that EF-G may in fact restrain the A-site tRNA, while maintaining the codon:anticodon interaction [150]. Both of these highly conserved and essential elongation factors bind to, and are inhibited by, ppGpp [151]. Indeed, EF-Tu can be inhibited either by direct competitive binding or by binding of ppGpp to a complex of EF-Tu and the cognate guanosine exchange factor EF-Ts to halt the EF-Tu cycle through kinetic trapping of this GTPase in an inactive complex [151]. Competitive inhibition of EF-G by ppGpp is also likely, as the use of an in vitro translation reconstitution system with a twofold excess of ppGpp over GTP, where EF-G was the limiting factor, revealed a twofold reduction in elongation rate [151].

Finally, upon tRNA binding at the ribosomal exit site, the release of the tRNA is catalysed by class 1 release factors including RF-1 and RF-2 following GTP hydrolysis. The class 2 release factor RF-3 is pre-associated with the ribosome and is responsible for catalysing the release of RF-1 and RF-2 to facilitate the next round of elongation [152,153]. Free RF-3 in the cytoplasm exists almost exclusively in the GDP-bound state, with a marked increase in ribosomal affinity when bound to GTP. In the ppGpp-bound state, it is hypothesised that the interaction of RF-3 with the ribosome would be negatively affected, supported by the fact that the recycling activity of RF-1 in a reconstituted assay decreased upon introduction of ppGpp into the system [153]. Furthermore, the reduction in the rate of exit of even a single tRNA can negatively influence the rate of translation of an entire polysome [154], suggesting that a slight reduction in RF-1 recycling could be more detrimental to total cellular translation than the reconstitution data suggests.

Overall, the cumulative inhibition of formation of the 30S pre-IC, aminoacyl-tRNA recruitment through inhibition of EF-Tu, translocation of tRNA through inhibition of EF-G and tRNA release and Type-1 release factor recycling through binding to RF3 results in a pronounced negative effect on the rate of translation upon induction of the stringent response.

## 9. Concluding Remarks

The innate complexity of 30S and 50S ribosome particle assembly makes it extremely unlikely that stochastic maturation will result in the correct conformation. The propensity for biological systems to enter the most energetically favourable state enhances the need for chaperone proteins responsible for ensuring correct folding of individual proteins, RNAs or, indeed, larger assemblies such as the prokaryotic ribosome. RA-GTPases fulfil this role during ribosome assembly, ensuring that the correct maturation level is achieved before either further r-protein recruitment or subunit joining takes place.

There are five major approaches that RA-GTPases use to facilitate ribosome assembly, each of which have been discussed here. 1: Direct non-r-protein regulatory factor recruitment (e.g., YbeY recruitment to the 16S rRNA by Era). 2: Quality control checkpoint before direct r-protein recruitment (e.g., RsgA monitoring the maturity of the decoding centre through interaction with h44; Era preventing S1 docking and 30S PreIC formation prior to 16S processing). 3: rRNA modulation to facilitate further r-protein joining (e.g., RbgA C-terminal domain interacting with 23S rRNA helices to protect and alter their conformation). 4: Disassembly of 70S particles with incorrect rRNA conformations to enable repair and resumption of translation (e.g., HflX-mediated splitting of heat-damaged 70S ribosomes). 5: Prevention of 70S assembly during unfavourable conditions as a consequence of the stringent response (e.g., HflX preventing subunit joining when bound to (p)ppGpp; increased mature 50S binding by ObgE during the stringent response). The combination of these results in a high level of control over the modular assembly pathway, enabling coupling of 70S assembly and translational activation to the fluctuating cellular GTP pool at any given time.

In addition to the longer-term inhibition of 70S assembly, the stringent response can inhibit the translational efficiency of both initiating and translating 70S ribosomes. This is achieved via targeting of the universally-conserved prokaryotic translation factors bIF-2, EF-Tu, EF-G and RF-3 [109,110,111,112], offering a mechanism of control for each of the major steps of translation: initiation, elongation and termination. All in all, the stringent response exhibits control over every aspect of the ribosomal life cycle, from the inhibition of rRNA synthesis by CodY [141], the inhibition of 16S rRNA processing [13], RA-GTPase-mediated 70S macroassembly [6,76] and, finally, to an increase in 70S splitting and degradation by HflX and ObgE [90,94,110,115].

The fact that ribosomes from every known organism require RA-GTPases for correct maturation and, thus, high efficiency in translation highlights the importance of these enzymes. The near-universal conservation of (p)ppGpp amongst prokaryotic organisms presents a novel and interesting target for antibiotic development; it has been long postulated that 70S ribosome assembly is a potentially powerful antimicrobial target [155]. Prevention of 70S assembly and maturation would result in severe reduction in bacterial viability, which may aid in the treatment of potentially multidrug-resistant infections in the years to come. While our understanding of ribosomal biogenesis at the molecular level is improving, it is still unclear what specific function these RA-GTPase enzymes have in ribosomal assembly. Additionally, despite the range of crystallographic and cryo-EM approaches that have been used to study prokaryotic RA-GTPases, the features that distinguish (p)ppGpp-binding RA-GTPases from non-(p)ppGpp-binding RA-GTPases remain elusive. No recognition motif or structural motif has been found to date that can enable the binding of (p)ppGpp, highlighting the need for continued on-going research in this field.
